# Efficacy and Safety of Spinal Collagen Mesotherapy in Patients with Chronic Low Back Pain in a Three-Month Follow-Up—Retrospective Study

**DOI:** 10.3390/jcm13030787

**Published:** 2024-01-30

**Authors:** Kamil Koszela, Marta Woldańska-Okońska, Robert Gasik

**Affiliations:** 1 Department of Neuroorthopedics and Neurology Clinic and Polyclinic, National Institute of Geriatrics, Rheumatology and Rehabilitation, 02-637 Warsaw, Poland; 2 Department of Internal Medicine, Rehabilitation and Physical Medicine, Medical University of Lodz, 90-419 Lodz, Poland

**Keywords:** local intradermal therapy (LIT), low back pain (LBP), myofascial pain syndrome (MPS), collagen type I, lignocaine

## Abstract

**Background**: Low back pain syndrome is associated with muscular and myofascial pain and is linked with muscle overstrain or a lack of regular physical activity as well as a habitual bad posture, which cause the overload of perispinal soft tissues. One of the forms of therapy of LBP is the mesotherapy of the spine, which consists of multi-point micro-injections of drugs or medicine mixtures, which include preparations of collagen type I. The aim of the study was to assess the efficacy and safety of mesotherapy with collagen type I. **Methods**: A retrospective analysis of the results of the treatment of patients with chronic low back pain syndrome using mesotherapy was performed. A total of 130 patients (83 women and 47 men; mean age: 51 ± 14 years) were divided into two groups: group I (*n* = 65), treated with collagen type I, and group II (*n* = 65), treated with lignocaine 1%. Mesotherapy was performed weekly over five weeks. Patients were assessed using the following scales: the VAS, Laitinen Scale, and Revised Oswestry Low Back Pain Disability Scale before the start of the treatment, after five treatments, and at the three-month follow-up visit. **Results**: A statistically significant improvement was observed after the use of spinal mesotherapy both with collagen type I and lignocaine, with the collagen treatment having better results at the three-month follow-up visit. No adverse effects were observed after the procedures. **Conclusions**: Spinal mesotherapy using collagen type I and lignocaine seems to be an effective method in the treatment of chronic LBP. Collagen mesotherapy gives better results in the long term. Mesotherapy is a safe form of therapy.

## 1. Introduction

Low back pain (LBP) is a very common health problem and a leading cause of disability worldwide [1]. Currently, over 90% of patients with LBP are not provided with a specific diagnosis, are managed inconsistently, and receive no specific preventive care; therefore, the number of patients in need of precise diagnostics is continuously increasing [2,3]. At present, back pain and musculoskeletal system dysfunction syndrome is becoming a lifestyle disease. It affects more and more people and is associated with, among other things, reduced physical activity, a habitually poor posture, and a sedentary lifestyle. The spine and surrounding tissues such as muscles, fascia, ligaments, and tendons are overloaded, which results in pain, increased tension, and reduced mobility [4].

In diagnosis and therapy, spine surgeons and orthopedic doctors usually focus on pain of spinal origin. However, many complaints are related to soft tissues, the treatment of which requires a different approach. Some authors recommend the direct injection of trigger points, which are hyperirritable palpable ‘knots’ in the taunt bands of the skeletal muscle fascia, present in some patients with muscular pain [5].

One of the methods for treating back pain is mesotherapy. Mesotherapy showed good results, reducing acute and chronic musculoskeletal pain, and, in addition, it is a well-tolerated treatment. This method is also called local intradermal therapy (LIT) [6].

Mesotherapy is a minimally invasive technique in which a liquid mixture of therapeutic ingredients (pharmaceutical and homeopathic medications, plant extracts, vitamins, etc.) is injected intra- or subcutaneously to treat local medical and cosmetic conditions [7]. The injections create microdeposits from which the drug is (or drugs are) slowly released into the underlying tissues. Because the medicine is administered near the intended site of action, mesotherapy has a rapid onset of action, a prolonged local effect, and a drug-sparing effect [8]. Before mesotherapy was first used in humans, it was utilized on a large scale in veterinary applications to treat the pain syndromes of the locomotor system in racehorses and dogs with a good therapeutic effect and safety profile. Those results encouraged the use of mesotherapy in the locomotor system in humans [9], and although it is still not so widely used in humans, this method has provided beneficial effects superior to systemic therapy in relieving musculoskeletal pain [10]. Mesotherapy has several benefits, one of which is the ability to provide a local pharmacological effect without the need for high systemic concentration. Additionally, it may have a dose-sparing effect since the intradermal delivery of active compounds in combination with other systemic therapies can work synergistically [11,12]. According to the current spinal mesotherapeutic protocols, the administration of a single drug is performed by local injections of small doses (0.1 mL of drug per point) with 30-gauge needles that are 3–4 mm in length (depending on the site of the procedure) [13,14,15,16]. Additionally, it has been suggested that the reflex effect caused by needles has clinical advantages for reducing pain [17,18]. Furthermore, the injection site could play a role in the analgesic effect, as reported in research papers concerning injections into trigger points in patients with chronic neck and low back pain. Trigger point injections were painful, but gave a satisfactory therapeutic effect [18,19]. Micro-injection stimulates receptors in the skin and subcutaneous tissue, causing micro-inflammation and the activation of repair mechanisms through the increase in inflammatory mediators. The stimulation of the circulatory, nervous, and immune systems improves blood flow through the tissues. Myofascial pain syndrome (MPS) is associated with an increased tension in the muscles and fascia, decreased physical activity, and thus blood stasis and congestion. Spinal mesotherapy affects the relaxation of tissues, and thus improves mobility. Therefore, one of the basic indications for mesotherapy is the pain originating from overloaded perispinal soft tissues [6,16,20]. It may be assumed that the rapid analgesic effect, often observed even after a single intradermal therapeutic application, and the analgesic effect induced by the medium- and long-term LIT are not only the result of micro-injections, the mechanical–chemical stimulus induced by the injected liquid, and the local pharmacological action, but are also the result of a series of complex interactions between the intradermal technique and dermal pain control systems. The dermis and, in particular, glial cells could be the new potential target of the drugs injected in the mesotherapy treatment. This mechanism of the effectiveness of mesotherapy, called mesodermal modulation, today represents the strongest rationale for applying mesotherapy in pain management [12,21,22]. In low back pain, the mesotherapy scheme consists of performing about 20 micro-injections. In the mesotherapy of the spine, diverse drugs can be used, such as collagen type I, lignocaine, NSAIDs, and others [16].

In our study, we used a medical device based on swine collagen (100 µg/2 mL vials), hypericum, NaCl, and water [23] injected via the mesotherapeutic technique. The medical device we use does not have randomized controlled studies, and our retrospective study represents the first systematic experience.

Lignocaine (lidocaine) is a local anesthetic drug that inhibits the formation and conduction of stimuli in nerve fibers. Lignocaine is completely absorbed from the injection site. Administered in the form of mesotherapy, it has a similar analgesic effect, reducing plasma concentrations of, e.g., IL-6, IL-8, and thus, is effective in the therapy of pain resulting from the pathological tension of the muscles and fascia. It is often used in spinal mesotherapy [16].

A recent systemic review of mesotherapy in medicine found that only a low percentage of identified studies had sufficiently solid methodology for their results to be considered. The authors also highlighted the need to compare a standardized mesotherapy protocol with systemic treatment [24], which justifies the topic of this publication.

The aim of the research was to assess the efficacy and safety of spinal mesotherapy with collagen type I versus lignocaine.

## 2. Materials and Methods

### 2.1. Study Design

Two groups of patients were distinguished based on a retrospective analysis of the medical records of patients who underwent spine mesotherapy with collagen type I (Group I) or lignocaine 1% (Group II). During their first visit, patients who had no contraindications to the treatment were offered mesotherapy treatment with collagen type I or lignocaine 1%. The doctor explained what mesotherapy is, what its advantages are, and how collagen/lignocaine mesotherapy works. The collagen mesotherapy treatment was more expensive. After the consultation, the patients chose one of the treatment options. Collagen mesotherapy was chosen by 65 patients out of 130.

Patients were treated from 1 January 2018 to 31 January 2023.

The study was approved by the Bioethics Committee for Scientific Research at Medical University in Lodz, number RNN/385/19/KE.

#### 2.1.1. Inclusion Criteria

– Pain localized in the triangle area between the posterior superior iliac spine and spinous process of L3, lasting more than three months and increasing during the deep palpation of the area of facet joints, 2–3 cm laterally from spinous processes (in the area of L3/L4 and/or L4/L5 and/or L5/S1;
– No motor or sensory radicular symptoms, which was confirmed in a neurologic examination;
– No lumbar stenosis;
– No other pathologies in the pelvis and lower limbs;
– No allergy to collagen type I or to lignocaine.

#### 2.1.2. Exclusion Criteria

– Lack of appropriate imaging diagnostics;
– Magnetic resonance imaging or computed tomography results (older than six months) confirming tumors, discitis, or spondylolisthesis;
– Pain associated with a systemic disease, e.g., rheumatoid arthritis, fibromyalgia;
– Taking painkillers and/or anti-inflammatory drugs during therapy;
– Prior low back surgery;
– Lack of consent of the patient and/or the guardian to examinations and participation in the program.

### 2.2. Study Protocol—Spinal Mesotherapy

**Figure 1:**
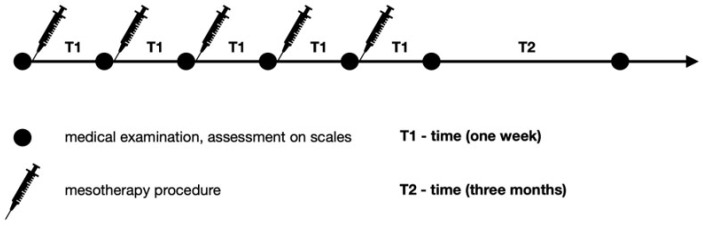
Diagnostic and therapeutic scheme.

Figure 1 presents a diagnostic and therapeutic scheme with a three-month follow-up. On the first day, a medical assessment was performed with the use of scales. Then, during an appointment, mesotherapy of the lumbosacral spine was performed. The procedure was performed weekly over five weeks. Before each mesotherapy procedure and at two control visits one week after the last treatment and three months later, the patients underwent physical examination. The patients received orthopedic and neurological examinations and assessments on scales. In the follow-up, adverse effects were assessed.

**Figure 2:**
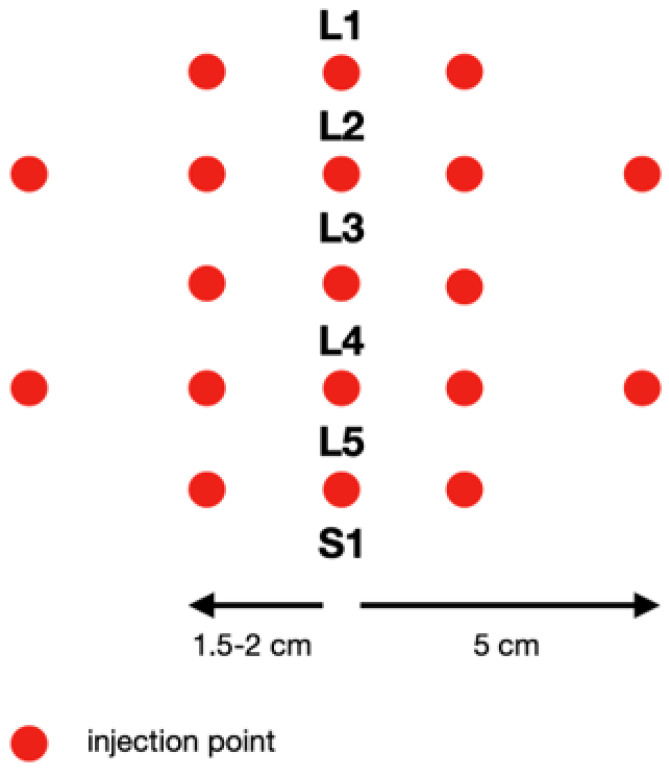
Scheme of injection of the lumbosacral spine.

In our study, one mesotherapy treatment consisted of about 20 micro-injections with about 0.1 mL of the drug (collagen type I or lignocaine 1%) per point. The type of preparation was determined at the first appointment. The scheme of the procedure is shown in Figure 2. The mesotherapy treatment was performed the same way by the same doctor in all patients. The drugs were injected to the skin in the following points: between the spinous processes and 1.5–2 cm and 5 cm laterally to the midline. This scheme was performed weekly over five weeks. For all patients, 30-gauge needles that were 0.3 mm in diameter and 12 mm in length were used.

### 2.3. Study Protocol–Assessment on Scales

Each patient was assessed using the Laitinen scale (0–16 points) [25], the VAS scale (0–10 points, where 10 is the maximum pain intensity) [26], and the Revised Oswestry Low Back Pain Disability Scale (0–50 points), which consists of ten items [27] graded from 0 to 5. The higher score on the ODI (Oswestry Disability Index) indicates the more severe disability caused by LBP. An assessment on all the above-mentioned scales was made on day 1 during the medical appointment before the mesotherapy treatment. Then, this assessment was repeated every week, before each mesotherapy treatment, and finally a week after the last mesotherapy treatment and then three months later. Assessments were performed in the afternoon. The evaluation scheme is presented in Figure 2.

### 2.4. Sample Size

Calculation of the sample size: to achieve a power of 80% with an alpha of 5% and a standardized effect size of 35%, each group should consist of at least 65 individuals.

### 2.5. Data Analysis

The statistical analysis was performed with R environment, version 4.3.1. The results were presented as the mean with the standard deviation and median with the interquartile range. The normality in the subgroups for continuous and quasi-continuous variables was assessed with the Shapiro–Wilk test. Non-parametric tests were used because of the non-normal distribution of almost all the subgroups. The ANOVA Kruskal–Wallis test was performed to assess at which point the treatment’s impact is significant. The ANOVA Friedman test and dedicated versions of the post hoc Nemenyi test were used to compare different time points. Post hoc power analysis was performed using G*Power 3.1.9.7 software. *p* < 0.05 was assessed as statistically significant.

## 3. Results

The retrospective study included 130 patients, consisting of 83 women and 47 men aged from 24 to 81 (Table 1) with chronic low back pain syndrome confirmed by physical examination and the following medical imaging tests: magnetic resonance (MRI) or computed tomography (CT). Patients were diagnosed and treated in a medical office. They all were examined by one orthopedist. Table 1 presents the parameters of the participants. There was no statistically significant difference in age (Mann–Whitney U test) and sex (chi2 test with Yates’ continuity correction).

A general linear model (GLM) was built for each pain scale to assess the effects of two different treatment methods at all time points and the interaction between time and the used treatment method. The Akaike information criterion (AIC) for each model was calculated. The results are settled in Table 2.

GLM revealed the impact of the time–medication interaction for the VAS and Oswestry scales. The difference between lignocaine and collagen at the end of the study is responsible for it, but the effect is feeble and should be treated carefully.

The majority of the variance in the whole group is explained by within factors, i.e., in time changes. The more complex the scale used, the lower the power of the ANOVA tests and the effect size (Table 3). The measurement error is more significant, i.e., for the VAS scale equals 1, Laitinen equals −2, and Oswestry Disability Index equals −3.16, and this is the leading cause of the smaller effect size and inadequate power for the Oswestry scale. The Akaike criterion allows one to choose between different models: the smaller the value, the better the model fitting to the data. The AIC rises with the complexity of the used scale, which is consistent with what was mentioned above. The VAS scale seems to be the best option for pain assessment during mesotherapy. Collagen and lidocaine comparisons are presented in Table 4.

Table 5 shows the side effects of mesotherapy in terms of the pain associated with the procedure. No adverse skin effects or other pathologies were observed in the short- and long-term follow-up. Additionally, it should be noted that with each subsequent treatment, a decrease in pain associated with the mesotherapy procedure using both collagen and lignocaine was observed.

## 4. Discussion

### 4.1. Discussion

Due to the fact that low back pain is reported by a growing number of patients, including increasingly younger ones, it is necessary to search for new therapeutic methods that will give a quick therapeutic effect and be effective and safe at the same time. Moreover, in musculoskeletal system pain syndromes, it is fundamental to eliminate the root cause of the pain (which is an often-forgotten fact). Taking into account the fact that in many cases, the low back pain syndrome has an overload origin and comes from perispinal soft tissues, it seems that the mesotherapy of the spine is one of such methods, as it not only has an analgetic function, but also affects the underlying cause of such conditions (i.e., causes the relaxation of affected regions). On the other hand, if LBP is caused by, e.g., symptomatic discopathy, with a spinal muscle spasm being a secondary reflex mechanism, mesotherapy can be used only as a symptomatic therapy, as the causal treatment of discopathy is completely different [16].

In the case of myofascial pain syndrome, when the use of mesotherapy muscle tone is normalized, mobility is improved and, as a result, pain is reduced. Furthermore, because there are still few studies describing collagen mesotherapy, our study compares the use of collagen with lignocaine.

Based on the obtained results, it can be concluded that the mesotherapy of the spine is an effective form of therapy for lumbosacral spine pain. On the VAS scale, one week after the last treatment, the pain was reduced from the average score of 7.7 to 2.7 in the case of collagen type I and from 7.5 to 2.5 in the case of lignocaine. After the three-month follow-up, the pain was reduced to 2.0 in the case of collagen type I, however, a slight increase to 3.0 was observed in the case of lignocaine. Similarly to the Laitinen scale, the score on the Revised Oswestry Low Back Pain Disability Scale was reduced both after five mesotherapy procedures in the case of collagen type I and lignocaine; however, a slight increase was observed in the case of lignocaine at the three-month follow-up. After 3 months of follow-up, a better therapeutic effect is observed after the use of type I collagen. This may be related to the repair and regeneration of tissues (muscles, fascia, ligaments), their relaxation, and improved mobility.

In earlier studies, similar results of the therapy were observed. In a randomized study, I. Akbas et al. [28] demonstrated the effectiveness of spinal mesotherapy in the treatment of low back pain in patients with lumbar disk herniation in comparison to systemic therapy. This condition can be associated with a spinal muscle spasm, which causes not only radicular but also muscular and myofascial pain as well as facet joint pain. Mesotherapy was performed in one group, whereas intravenous dexketoprofen was administered to the control group. In this study, patients were assessed using the VAS scale.

In a retrospective observational study, G. Ronconi et al. [29] found that diclofenac mesotherapy appeared to be a viable treatment to reduce pain and improve function in patients affected by chronic moderate-to-severe nonspecific low back pain. As in our study, patients underwent five mesotherapy sessions with a 12-week follow-up. Additionally, to assess the efficacy of the therapy, similar scales were used. A pain Visual Analogue Scale primary outcome, Oswestry Disability Index secondary outcome, and the Short-Form McGill Pain Questionnaire were used.

In a pilot study, P. Godek [30] observed an improvement with all three used techniques of collagen type I administration. The subcutaneous administration of collagen was not inferior in terms of effectiveness to periradicular and epidural injections in the treatment of LBP in lumbar spondylosis. Patients were assessed similarly as in our study, using the VAS, Oswestry Scale, and Laitinen Scale. The main differences with the present study were the injection technique, the depth of the injection, and the fact that collagen type I was not compared with any other drug.

In a randomized, single-blind controlled trial, A. Nitecka–Buchta et al. [31] confirmed that the intramuscular injection of collagen type I was a more efficient method for reducing myofascial pain within masseter muscles than the intramuscular injection of lignocaine, just as in our study. On the other hand, the patients were assessed using only the VAS scale with a shorter follow-up.

In the next short-term randomized controlled trial, A. Di Cesare et al. [18], the effects of trigger point mesotherapy and acupuncture mesotherapy were compared. The following were used for the assessment: the VAS scale, verbal rating scale (VRS), McGill Pain Questionnaire Short Form (SFMPQ), Roland Morris Disability Questionnaire (RMQ), and Oswestry Low Back Pain Disability Questionnaire (ODQ). Their results suggested that the response to acupuncture mesotherapy could be greater than the response to trigger point mesotherapy. Similarly to our study, acupuncture points were also used to apply mesotherapy [18].

In their study, B. Bifarini et al. [32] confirmed that in mesotherapy, even a lower dose of drugs could induce a clinically useful result. They also concluded that the useful effect of mesotherapy was only partly caused by the pharmacological action. Of importance are also the injection technique and the activation of the therapeutic mechanisms arising after the injection. A therapeutic effect was observed in both groups treated with a full drug dose and with a half dose, which suggests that it was associated both with the administered preparation and with the injection technique, i.e., with spinal mesotherapy.

Furthermore, mesotherapy is also used in emergency medicine. In another randomized controlled trial, A. O. Kocak [10] administered intravenous dexketoprofen in the control group and performed mesotherapy treatment in the other group. Patients were assessed using the VAS scale and the conclusion was that it is possible for topical mesotherapy to be more effective than systemic therapy for pain relief. This study showed that mesotherapy could be effective not only in chronic pain syndromes, but also in acute ones, which means that spinal mesotherapy could become a new therapeutic tool in emergency medicine and the superficial spinal injuries of a muscular or myofascial nature. Therefore, more research should be conducted in this direction.

In our study, there were no serious complications after the use of the collagen or lignocaine mesotherapy of the lumbar spine. Similarly, in other studies, mesotherapy seemed to be a safe form of treatment of musculoskeletal pain syndromes [24,33,34]. On the other hand, we observed that some patients experienced pain associated with the mesotherapy treatment. It was seen especially in patients experiencing strong pain in the perispinal soft tissues on palpation. Ailments subsided a few hours after the procedure. With each subsequent treatment, the pain was reduced, the tense soft tissues (muscles, fascia) became relaxed, and thus, the mobility of the spine was improved.

Data from the study by S. Brauneis et al. [8] indicate that the active ingredients which infiltrated deep into the skin induce an intradermal response between those ingredients and the neural and cellular structures of the skin, resulting in the typical medication-saving effect of mesotherapy. This aspect of the treatment, together with a few systemic adverse effects, is extremely important for both the patients and the public health service. Although further research is needed to determine how to integrate mesotherapy in different clinical settings, the researchers found it to be a useful technique that is accessible to a practicing clinician. As the authors point out, the research described may also be useful in carrying out future clinical trials.

It should be emphasized that spinal mesotherapy was recommended by the Italian Mesotherapy Society (SIM). However, this recommendation applied to the injection of only those drugs or products which were chosen based on the diagnosis and authorized indications, thus avoiding the risk of mixing multiple products without evidence of their efficacy and safety [35].

### 4.2. Limitations of the Study

It would be advantageous if our study could be conducted with a saline control group, but due to its retrospective nature, this was not an option. Therefore, it would be worth conducting a randomized study, which additionally should involve a larger group of patients and a longer follow-up period. Furthermore, during the three-month follow-up, some of the patients could have been subjected to ad hoc analgesic pharmacotherapy, not to overload the spine, e.g., during work, which could have affected the results. Moreover, it is possible for the BMI to influence the therapy results, specifically via the absorption rate of the collagen type I or lignocaine. It would be advisable to include this parameter in the next study, e.g., by assessing in a USG examination the thickness of the subcutaneous fat tissue in the lumbar area. Furthermore, the study did not include patients regularly taking analgesics or anti-inflammatory and myorelaxant drugs, neither those being on sick leave nor undergoing physiotherapy during this study period.

## 5. Conclusions

Both collagen type I and lignocaine provide a therapeutic, analgesic effect in the treatment of chronic LBP. Based on the performed analysis, it can be concluded that using collagen mesotherapy has a better effect in the long term. Our research confirmed that spinal mesotherapy is a safe therapy for chronic low back pain, particularly muscular pain. However, this method requires further research on a larger group of patients with a longer follow-up period.

## Figures and Tables

**Table 1:** Participant characteristics.

Study Groups	Spinal Mesotherapy			
	A Collagen (*n* = 65)	%	B Lignocaine 1% (*n* = 65)	%
Women	44	68%	39	60%
Men	21	32%	26	40%
Age [minimum]	24		27	
Age [maximum]	79		81	
Age [mean, SD]	51 ± 13.88		52 ± 14.01	

**Table 2:** Scale scores at study time points.

	Collagen Group (*n* = 65)		Lidocaine Group (*n* = 65)		GLM *p*-Values			
	Mean ± SD	Median (IQR)	Mean ± SD	Median (IQR)	Time	Treatment	Interaction Time–Treatment	AIC
VAS-before therapy	7.7 ± 0.9	8 (7–8)	7.7 ± 0.9	8 (7–8)	<0.001	0.263	0.005	1486.3
VAS-after therapy	2.7 ± 1.3	3 (2–3)	2.5 ± 1.0	2 (2–3)				
VAS-three-months follow-up	2.0 ± 1.1	2 (1–3)	3.0 ± 1.2	3 (2–4)				
Laitinen Scale-before therapy	11.0 ± 1.7	11 (10–12)	10.9 ± 1.5	11 (10–12)	<0.001	0.167	0.119	1735.3
Laitinen Scale-after therapy	3.7 ± 1.7	4 (3–5)	2.9 ± 0.8	3 (2–3)				
Laitinen Scale-three-months follow-up	2.6 ± 1.2	3 (2–3)	3.3 ± 1.1	3 (3–4)				
Revised Oswestry Low Back Pain Disability Scale-before therapy	30.6 ± 3.0	31 (28–32)	30.2 ± 4.2	31 (28–32)	<0.001	0.183	0.023	2501.4
Revised Oswestry Low Back Pain Disability Scale-after therapy	9.6 ± 3.5	9 (7–11)	8.4 ± 3.2	8 (6–10)				
Revised Oswestry Low Back Pain Disability Scale-three-months follow-up	6.4 ± 3.1	6 (4–8)	9.3 ± 3.4	9 (7–11)				

**Table 3:** The influence of the pain scale choice on the study results and their interpretation.

VAS	ANOVA for Repeated Measures		
	Treatment	Time	Time–Treatment
% of explained variance	0.40	98.50	1.14
power	0.108	1.0	0.127
effect size	0.051	0.369	0.039
**Laitinen Scale**	**ANOVA for Repeated Measures**		
	**Treatment**	**Time**	**Time–Treatment**
% of explained variance	0.009	99.7	0.290
power	0.051	0.999	0.142
effect size	0.007	0.253	0.043
**Revised Oswestry Low Back Pain Disability Scale**	**ANOVA for Repeated Measures**		
	**Treatment**	**Time**	**Time–Treatment**
% of explained variance	0.040	99.40	0.550
power	0.058	0.612	0.052
effect size	0.018	0.091	0.007

**Table 4:** Post hoc Nemenyi tests for Kruskal–Wallis ANOVA (collagen and lidocaine comparisons) and for Friedman ANOVA (point to point comparisons for each treatment separately).

VAS						
	Collagen-before Therapy	Collagen-after Therapy	Collagen-Three-Months Follow-Up	Lidocaine-before therapy	Lidocaine-after Therapy	Lidocaine-Three-Months Follow-Up
Collagen-before therapy	-	<0.001	<0.001	1.0	-	-
Collagen- after therapy	<0.001	-	0.047	-	0.986	0.974
Collagen-three-months follow-up	<0.001	0.047	-	-	0.577	0.028
Lidocaine-before therapy	1.0	-	-	-	<0.001	<0.001
Lidocaine-after therapy	-	0.986	0.577	<0.001	-	0.19
Lidocaine-three-months follow-up	-	0.974	0.028	<0.001	0.19	-
**Laitinen Scale**						
	**C** **ollagen-before Therapy**	**C** **ollagen-after Therapy**	**C** **ollagen-Three-Months Follow-Up**	**L** **idocaine-before Therapy**	**L** **idocaine-after Therapy**	**Lidocaine-Three-Months Follow-Up**
Collagen-before therapy	-	<0.001	<0.001	1.0	-	-
Collagen-after therapy	<0.001	-	0.0045	-	0.114	0.810
Collagen-three-months follow-up	<0.001	0.0045	-	-	0.992	0.444
Lidocaine-before therapy	1.0	-	-	-	<0.001	<0.001
Lidocaine-after therapy	-	0.114	0.992	<0.001	-	0.25
Lidocaine-three-months follow-up	-	0.810	0.444	<0.001	0.25	-
**Revised Oswestry Low Back Pain Disability Scale**						
	**C** **ollagen-before Therapy**	**C** **ollagen-after Therapy**	**C** **ollagen-Three-Months Follow-Up**	**L** **idocaine-before Therapy**	**L** **idocaine** **-** **after Therapy**	**Lidocaine-Three-Months Follow-Up**
Collagen-before therapy	-	<0.001	<0.001	0.999	-	-
Collagen-after therapy	<0.001	-	0.013	-	0.730	-
Collagen-three-months follow-up	<0.001	0.013	-	-	0.237	0.998
Lidocaine-before therapy	0.999	-	-	-	<0.001	0.011
Lidocaine-after therapy	-	0.730	0.237	<0.001	-	0.059
Lidocaine-three-months follow-up	-	0.998	0.011	<0.001	0.059	-

**Table 5:** Side effects of spinal mesotherapy.

	Spinal Mesotherapy			
	Collagen (*n* = 65)	*n* (%)	Lignocaine 1% (*n* = 65)	*n* (%)
Pain during first mesotherapy procedure (VAS ≥ 7)	Yes	2 (3.0)	Yes	1 (1.5)
Pain during first mesotherapy procedure (VAS 4–6)	Yes	9 (13.8)	Yes	6 (9.2)
Pain during first mesotherapy procedure (VAS < 4)	Yes	30 (46.2)	Yes	21 (32.3)
Pain during 5th mesotherapy procedure (VAS ≥ 7)	No	0 (0)	No	0 (0)
Pain during 5th mesotherapy procedure (VAS 4–6)	Yes	3 (4.6)	Yes	4 (6.2)
Pain during 5th mesotherapy procedure (VAS < 4)	Yes	10 (15.4)	Yes	7 (10.8)
Fever after first mesotherapy procedure	No	0 (0)	Yes	1 (1.5)

## Data Availability

The data are available from the corresponding author if required.
